# Phosphaturic Mesenchymal Tumors with or without Phosphate Metabolism Derangements

**DOI:** 10.3390/curroncol30080541

**Published:** 2023-08-08

**Authors:** Andrea Montanari, Maria Giulia Pirini, Ludovica Lotrecchiano, Lorenzo Di Prinzio, Guido Zavatta

**Affiliations:** 1 Orthopaedics and Traumatology Unit, IRCCS Azienda Ospedaliero-Universitaria di Bologna, 40138 Bologna, Italy; 2 Pathology Unit, IRCCS Azienda Ospedaliero-Universitaria di Bologna, 40138 Bologna, Italy; 3 Radiology Unit, IRCCS Azienda Ospedaliero-Universitaria di Bologna, 40138 Bologna, Italy; 4 Division of Endocrinology and Diabetes Prevention and Care, IRCCS Azienda Ospedaliero-Universitaria di Bologna, 40138 Bologna, Italy; 5 Department of Medical and Surgical Sciences (DIMEC), Alma Mater Studiorum University of Bologna, 40138 Bologna, Italy

**Keywords:** phosphaturic tumor, tumor-induced osteomalacia, FGF-23, phosphatonin, surgery, orthopedics, TIO, rare bone disease

## Abstract

Phosphaturic mesenchymal tumors (PMT) are rare neoplasms, which can give rise to a multifaceted syndrome, otherwise called tumor-induced osteomalacia (TIO). Localizing these tumors is crucial to obtain a cure for the phosphate metabolism derangement, which is often the main cause leading the patient to seek medical help, because of invalidating physical and neuromuscular symptoms. A proportion of these tumors is completely silent and may grow unnoticed, unless they become large enough to produce pain or discomfort. FGF-23 can be produced by several benign or malignant PMTs. The phosphate metabolism, radiology and histology of these rare tumors must be collectively assessed by a multidisciplinary team aimed at curing the disease locally and improving patients’ quality of life. This narrative review, authored by multiple specialists of a tertiary care hospital center, will describe endocrine, radiological and histological features of these tumors, as well as present surgical and interventional strategies to manage PMTs.

## 1. Introduction

Phosphaturic mesenchymal tumors (PMT) are rare neoplasms, which can give rise to a multifaceted syndrome, otherwise called tumor-induced osteomalacia (TIO). TIO is extremely rare and epidemiology on this condition is currently unknown. Approximately 450 PMTs have thus far been reported in the literature [1]. The skeleton is affected in its whole by the biological consequences of small proteic substances secreted by PMTs, which usually localize within the bone or in the soft tissue. The peptides secreted by these tumors, called phosphatonins, cause the kidneys to lose more phosphate than usual, thus leading to demineralization of the skeleton, fractures or pseudofractures [2]. FGF-23 is the best known phosphatonin to date. Localizing these tumors is crucial to obtain a cure for the phosphate metabolism derangement, which is often the main reason for the patient to seek medical help, because of invalidating physical and neuromuscular symptoms. Of note, phosphate is a mainly intracellular anion, which serves as a key mediator in many physiological processes.

To make things even more complicated, a proportion of these tumors is completely silent and may grow unnoticed, unless they become large enough to produce pain or discomfort. FGF-23 can be produced by several benign or malignant lesions. In fact, TIO can be caused by tumors other than PMTs, such as odontogenic fibroma, hemangiopericytomas, giant cell tumor of tendons and, in a minority of cases, phosphaturic malignant tumors. Although the histology of these tumors is usually benign [3], local aggressiveness must be considered, possibly causing multiple recurrences after first resection.

The phosphate metabolism, radiology and histology of these rare tumors must be assessed by a multidisciplinary team aimed at curing the disease locally, as well as improving patients’ quality of life. This narrative review, authored by multiple specialists of a tertiary care hospital center will describe endocrine, radiological, histological and surgical strategies to manage PMTs.

This manuscript is a narrative review of the literature on TIO and PMTs. The aim of this review was to collect and merge information on clinical characteristics, pathology, imaging and treatments of these rare conditions. Each author separately examined the literature of the last 40 years about TIO and PMTs, according to their expertise and medical background.

## 2. Clinical Characteristics

PMTs equally affect men and women. Suspicion is higher in the 4th or 5th decades of life. Time to diagnosis varies widely, with often delayed diagnoses up to 4 to 7 years [4,5,6]. TIO can also occur in children or adolescents, often resembling rickets, with gait and skeletal abnormalities as well as growth retardation [7,8]. A systematic review in 2022 analyzed 468 articles covering 895 cases of TIO [9]. These authors found that the median age of presentation was 46 years, with a range from 9 months to 90 years. Out of 754 surgically treated PMTs, 279 had follow-up for at least 6 months. Of these patients, 14.2% reported disease recurrence. Among recurrent tumors, 20% were malignant. Most tumors are found in the tendons of the lower limbs (46.4%), followed by the head and neck (25.7%) and the pelvic area (10.3%).

PMTs most often produce factors called phosphatonins, which are able to cause phosphate wasting and are also capable of decreasing production of 1,25(OH)_2_ vitamin D, which is the active form of vitamin D3. This derangement, along with phosphate depletion, causes the skeleton to progressively demineralize, even despite treatment with vitamin D3 supplements [10], because FGF-23 excess inhibits vitamin D activation. These tumors may oversecrete FGF-23, MEPE, sFRP4, FGF-7 and many other phosphatonins which are still unknown. Biochemical severity correlates with tumor size. FGF-23 levels are highest in tumors larger than 5 cm. Symptoms may vary from muscle weakness/fatigue, bone pain, trouble walking to pathological or stress fractures, leading to reduced height due to spinal deformities. Performing laboratory workups is important to unveil a metabolic alteration.

Phosphate levels should be interpreted according to age, especially in young adults where serum phosphate is physiologically higher, and phosphate depletion could be easily overlooked. Hospitalized patients with hypophosphatemia usually show poor outcomes [11]. Low serum phosphorus is seen in up to 1/20 hospitalized patients [12]. The small intestine can absorb 60–65% of dietary phosphate, thanks to the sodium-dependent phosphate cotransporter type IIb (NaPiIIb). When dietary load increases, intestinal absorption increases also, with consequent higher demand for phosphate excretion through the kidneys [13].

**Figure 1:**
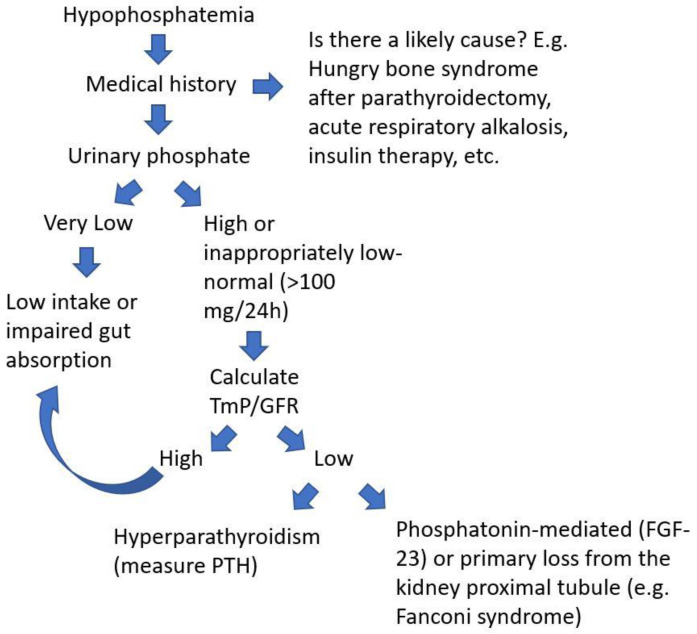
Flow-chart to evaluate a patient with low serum phosphate to suspect a diagnosis of a PMT.

On suspicion of PMT, the clinician should bear in mind that 80% of filtered phosphate is reabsorbed in the proximal tubule and that when hypophosphatemia is diagnosed, a 24 h urinary phosphate >100 mg can be used to suspect renal leak of phosphate. A recent review summarizes the diagnostic algorithm of hypophosphatemia and when to consider TIO based on laboratory parameters [14]. Briefly, two calculations can be easily made to figure out if phosphate is lost through the kidneys. First, fractional excretion of phosphorus (FEP) which is calculated using the following formula: FEP = (urinary phosphate × serum creatinine)/(serum phosphate × urinary creatinine). In the case of hypophosphatemia, an FEP greater than 5% is highly suspicious for urinary phosphate loss. Another important parameter derived from standard biochemistries is the TmP/GFR. TmP stands for the maximum tubular absorption of phosphate, and it depends on renal function. When serum phosphate increases, if kidney function is normal, phosphate reabsorption in the nephron is coherently diminished, thus keeping homeostasis. And vice versa, when serum phosphate is low, the kidneys try to reabsorb phosphate as much as possible. Physiologically, approximately 80% of phosphorus filtered through the kidneys is efficiently reabsorbed in the proximal tubule of the nephron. This means that when phosphorus is low, the TmP should be much higher than 80% [15]. In other words, when TmP/GFR is low (<0.80) despite low serum phosphate, the kidney is then wasting phosphate. This may be due to a disease of the kidney tubule or in the setting of a PMT secreting phosphatonins. Figure 1 presents a clinical flowchart to evaluate a patient with hypophosphatemia.

Alkaline phosphatase is usually elevated because of the underlying defect of mineralization of the osteoid [16,17], as in most other causes of osteomalacia [18]. Typically, other bone turnover markers are elevated and tend to further increase after surgical removal of the offending tumor [19], because of acute re-mineralization of osteoid tissue. Normalization of bone turnover markers might occur only after several months. Other endocrine parameters may vary. For example, because of a chronic inhibition of 1,25(OH)_2_ vitamin D, hyperparathyroidism may gradually develop, although serum calcium and creatinine are expected to be within normal limits (Table 1). Reduced synthesis of calcitriol leads to a reduced intestinal absorption of calcium, thereby causing a negative calcium balance which causes elevation in parathyroid hormone and often secondary hyperparathyroidism. Secondary hyperparathyroidism might also be due to the lack of inhibitory action of calcitriol on the parathyroid glands [20]. Intestinal absorption of phosphate is also reduced due to low calcitriol levels, thus further contributing to the pathophysiology of hypophosphatemia [21]. A correct interpretation of all these biomarkers, both during the diagnostic assessment and after surgical removal of the tumors might help both to accelerate the diagnosis and to detect early recurrence.

The phosphaturic hormone FGF-23, is usually elevated [22], and it usually decreases after complete resection of the tumor [23]. However, in some circumstances, FGF-23 levels might be found to be within normal limits, challenging the diagnosis. For example, blood draws from peripheral veins might return normal values, while measuring FGF-23 in the vein in proximity of the suspected causative tumor might produce quite different results, with elevated FGF-23 levels in the vein draining from the tumor [24]. Other phosphatonins might also be elevated with less significant FGF-23 elevations. For example, FGF-7 may be co-secreted with FGF-23. A case showed a much higher secretion of FGF-7 in comparison with FGF-23, which enabled the clinician to correctly localize the FGF-7 producing tumor [25] through an FGF-7 gradient rather than an FGF-23 gradient.

Normal serum FGF-23 levels should prompt the clinician to question the actual phosphatonin causing TIO. Secreted frizzled-related protein 4 (Sfr4), matrix extracellular phosphoglycoprotein (MEPE), as well as FGF-7 are other rare non-FGF-23 phosphatonins to be considered [26,27].

Under a molecular perspective, pathogenesis of FGF-23 production and regulation in PMTs has become clearer over the past 10 years. Lee et al. discovered that most PMTs express fusion genes between fibronectin (FN1) and FGFR1 [28,29], which are genes found on chromosome 2 and chromosome 8, respectively, and generate a new ‘chimeric’ receptor. The resultant chimeric protein FN-FGFR1 increases substantially, because the FN1 promoter is included in the new fused gene and because the FN1 promoter is constitutively active, thereby generating more ‘chimeric’ proteins. The overall increased signaling of these new aberrant receptors leads to FGF-23 overexpression, which further enhances its production by stimulating the chimeric receptors in an autocrine/paracrine fashion. Of note, these new chimeric receptors seem to function independently of Klotho co-receptor, as opposed to FGFR1 ‘naïve’ receptors [21].

Bone mineral density measured by dual-energy X-ray absorptiometry (DXA) is usually decreased, thus explaining a deficient mineralization. In a recent systematic review, 62% of patients with PMTs and with available BMD (Bone Mineral Density) data, had a BMD T-score of the lumbar spine ≤−2.5 (*n* = 61/99). In total, 346 of 895 patients (39%), reported at least one fracture [9].

The clinician should always bear in mind a few genetic conditions can mimic TIO or vice versa. For example, late-onset autosomal dominant hypophosphatemic rickets (ADHR) might overlap with TIO because the age of onset of this kind of rickets is usually not within the first years of life, as opposed to other forms of genetic rickets. Impaired growth and leg bowing, however, are usually signs of genetic rickets, as opposed to TIO where development of the skeleton is generally unaffected. Also, family history and the onset of hypophosphatemia in childhood might be other helpful clues in the differential diagnosis. Renal Fanconi syndrome usually requires added laboratory work-up with assessment of urine glucose and urine protein to confirm the suspicion of a tubular defect. Genetic consultation should also be a consideration in patients with negative imaging to definitely rule out genetic forms of hypophosphatemia [30].

## 3. Imaging and Radiological Features of PMTs

Most cases of TIO are caused by PMTs, and are usually due to excess FGF-23. Locations are odd, making them often difficult to detect by radiologists. PMTs are found in the bone or soft tissues [31]. Lower extremities and head and neck are the two commonest sites [32]. Roughly half of cases (53%) occur in the bone, the other remaining cases in the soft tissue (45%) and in the skin (2%) [33,34].

Nuclear medicine imaging has been suggested to be the most accurate method to locate these tumors. For example, technetium bone scintigraphy, Gallium-68 DOTATATE and DOTANOC PET can be used as first-line functional tools to locate the tumors in a whole body scan [35]; one of these studies suggested also that Ga-DOTATATE PET/CT had a greater sensitivity and specificity, useful for the localization of PMTs in TIO [36], which can be a difficult diagnostic point due to variety of presentation and localization of this heterogeneous pathology.

In the field of oncology, Whole-Body MRI (WB-MRI) is also increasingly being used for the detection of metastatic bone tumors or lymph nodes metastasis in more centers. Coronal STIR sequences along with T1w sequences are useful in finding areas of bone edema secondary to fracture, while the use of DWI (Diffusion-Weighted Imaging) is still controversial and to be further investigated for this group of tumors [37]. It would be useful to conduct an investigation to evaluate the value of DWI in PMT diagnosis and characterization, as well as to examine the relationship between DWI findings and histological characteristics, particularly in distinguishing benign from malignant PMTs.

While the majority of the published literature on imaging studies of PMTs is focused on the choice of the best modality in localizing the tumors, little literature is available on the imaging features of PMTs masses.

For a better characterization of the lesion, after localization, CT scan can be easily performed. If the tumor is localized in soft tissues, on CT scans, the tumor exhibits a round or oval, well-bordered, isodense/hypodense soft tissue mass and displays a uniform enhancement when the tumor is small [31]; instead, bone lesions appear as typical osteolytic areas, show a narrow zone of transition and commonly contain internal matrix [1]. The disadvantage of CT lies in the use of ionizing radiation.

MRI plays an important role in lesion characterization and does not involve ionizing radiation. Considering the different histopathological variants of the tumor, the variant affecting mixed connective tissue (PMT-MCT), can present a typical MR appearance. On T1-weighted imaging, the tumor can be isointense compared to muscle, while on T2-weighted imaging the tumor is usually hyperintense. Consistent homogenous enhancement on post contrast T1w fat-suppression imaging is usually seen. However, it is necessary to consider that size can alter the signal and characteristic of the lesion. On the one hand, small PMTs might display homogeneous signal intensity both on T1w and T2w and uniform enhancement after T1wI contrast. By contrast, large tumors may show more heterogeneity on T2w, T1w, as well as on post-contrast T1w images. Heterogeneous low signals derive from vascular flow voids [31], or from matrix [38]. Differential diagnosis should be made with any soft tissue mass, including neurofibroma, hemangioma, leiomyoma, giant cell tumor, hemangioendothelioma, fibroma, neurofibrosarcoma. When PMTs are within the bone, any focal bone lesion such as solitary fibrous tumors, fibrous dysplasia or giant cell tumors should be considered in the differential diagnosis [1]. As such, clinical presentation and laboratory correlation are critical to PMT recognition and accurate diagnosis.

Regarding functional imaging, octreotide-based radiotracers appear to show the greatest advantages in detecting PMTs in comparison with other commonly used radiotracers such as 18F-fluorodeoxyglucose (^18^F-FDG). This is because PMTs express the somatostatin receptor (SSTR). ^68^Ga DOTATATE demonstrates high affinity for somatostatin receptor 2 (SSTR2), which is highly expressed in these tumors. ^68^Ga DOTATATE positron emission tomography (PET) combined with computed tomography has been suggested to be a better radioisotope than ^18^F-FDG-CT, because some PMTs do not take up ^18^F-FDG. ^68^Ga-DOTATATE PET/CT demonstrated may be the best single study for localization of PMTs in TIO. Importantly, the imaging procedure must be performed from head to toe, to also include body extremities. This should be specifically demanded from the technician acquiring the images [35,36].

It would be useful to conduct a large prospective and multicenter investigation to establish which imaging approach is the most sensitive and specific for early identification and exact localization, by comparing different imaging modalities.

For now, the clinician should consider the advantages of integrating different imaging techniques, such as PET/CT, MRI, and CT to localize PMTs accurately. Multicenter studies with centralized imaging could be performed to define the best imaging technique/s in TIO.

## 4. Surgery and Non-Surgical Alternatives

Surgical treatment of PMTs requires the complete removal of the tumor and of any surrounding tissue that may be affected. This should be theoretically done over a wide margin to ensure complete resection. It is critical to have a surgical margin which is wide enough, as these tumors have been known to recur [39,40,41]. However, considering the benign characteristics of these tumors, it is still debated on how wide margins should be, in order to reduce the risk of recurrence, minimizing the morbidity of surgeries especially in particular anatomic sites such as the spine. Almost always, tumor resection represents a cure, and there is a relatively rapid improvement of symptoms, if any, following complete tumor removal. FGF-23 disappears quickly from circulation with a half-life of around 45 min [42]. Within five days after surgery, most patients show a return of serum phosphate to normal levels, but sometimes this may take up to several days. In children, phosphate was seen to return to normal serum levels within 2 days. In fact, the confirmation of TIO diagnosis is possible upon observing serum phosphorus levels returning to normal after tumor resection. Patients usually feel better in days to weeks following tumor removal. Bone healing starts immediately, although this might also depend on the severity of the disease. It may take up to one year or more to observe significant clinical improvement [43].

Radiofrequency ablation (RFA) has been reported as a potential treatment method [44]. RFA is a minimally invasive technique that has been used for the treatment of various bone tumors, including some cases of phosphaturic tumors. It involves the use of radiofrequency energy in order to heat and destroy cancerous tissue. During the procedure, a small needle-like probe is inserted into the tumor under the guidance of imaging techniques such as ultrasound or computerized tomography (CT) scans. Once the probe is in place, it emits high-frequency energy that generates heat and destroys tumor cells. RFA has several potential benefits for the treatment of phosphaturic tumors. Firstly, it is a minimally invasive technique that can be performed on an outpatient basis, which means that patients may experience less pain, fewer complications and faster recovery times compared to traditional open surgery. Secondly, RFA can be used to treat tumors that are difficult to remove surgically due to their location or proximity to vital organs. Sutcliffe et al. reported on a small case series (four patients) in which RFA was successfully used to treat PMTs. The study reported that RFA was able to achieve complete tumor ablation in all four cases without any significant complication [45]. Another case report by Li et al. showed that percutaneous RFA was a safe and effective procedure for a patient with a PMT [46]. However, there are also some potential limitations of RFA for the treatment of PMTs. One concern is that the procedure may not be as effective for larger tumors or tumors that have spread to other areas of the body. Additionally, there is a potential risk of complications such as bleeding, infection or damage to surrounding tissues. In conclusion, RFA has shown itself to be promising as a minimally invasive treatment choice for some cases of PMTs. However, each patient’s case should be evaluated individually, and the risks and benefits of RFA must be carefully weighed against other available treatment options. More research is needed to determine the long-term outcomes and efficacy of RFA for the treatment of PMTs.

Although rare, late recurrence due to metastatic disease is a possibility. It is estimated to happen in <5% of TIO patients [40,47]. Malignant PMTs commonly metastasize to the lungs. In that case, lesions may be too small to be visualized, therefore high-resolution CT is recommended. A miliary pattern might also be present. The prognosis after metastasis varies significantly, and there have been reports of patients surviving for up to 30 years [48].

## 5. Histology

Evans and Azzopardi first saw that TIO-associated mesenchymal tumors might represent distinct neoplasms. This happened in 1972. It was in 1987 when the term “phosphaturic mesenchymal tumor, mixed connective tissue variant” was then coined [49]. In other words, while in the past decades it was thought that virtually all mesenchymal tumors (e.g., osteosarcoma, hemangiopericytomas, giant cell-rich tumors of bone or osteoblastoma) could occasionally give rise to TIO, in the last decade it has become clearer that PMTs which cause TIO represent a unique entity, which is the reason why these neoplasms are now acknowledged with a definitive nomenclature: PMT [1].

**Figure 2:**
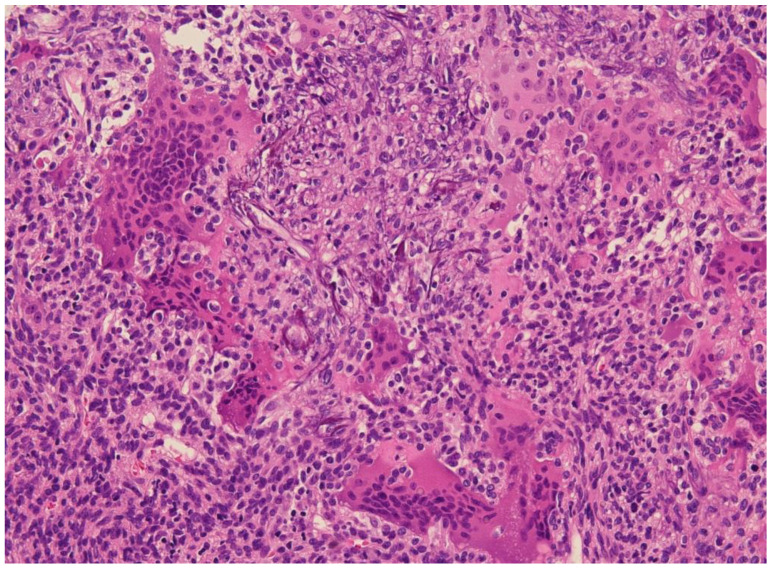
E&E 20×. Phosphaturic mesenchymal tumor with grungy calcifications and fibrohistiocytic areas containing osteoclast-type giant cells similar to giant cell tumors of bone.

Diagnosis has often been restricted to cases associated with tumor-induced osteomalacia (TIO) and, hence, diagnosis of “non-phosphaturic variants” remains challenging. So-called non-phosphaturic PMTs (i.e., hormonally inactive) in most instances are superficial PMTs found before the diagnosis of osteomalacia. This may be because osteomalacia has not been recognized clinically or because these tumors produce fewer phosphatonins, or because there might be an underlying compensatory mechanism. Microscopically, these tumors are prone to infiltrating surrounding tissues, and are difficult to resect with negative margins [1,47]. Fibroblastic-like spindled or stellate undifferentiated mesenchymal cells set irregularly into prominent sclerotic matrix with characteristic bluish to hyaline smudgy/grungy appearance (Figure 2). The tumor almost always has vascular proliferation which often leads to a diagnosis of sclerosing hemangioma or hemangiopericytoma (HPC-like vascular pattern). Currently, PMTs comprise tumors without HPC-like vessels or osteoclasts, cytologically banal neoplasms with possibly worrisome aspects (e.g., osteoid-like matrix), and frankly sarcomatous tumors. In some instances, the tumor is characterized by unusual features where oxalate-like crystals and mature cartilage largely obscure the neoplastic nature of the lesion. Other variants of PMTs include chondromyxoid fibroma-like, osteoblastoma-like, giant cell granuloma-like and angiomyolipoma-like. Most PMTs express FGFR1, CD56, ERG, SATB2 and somatostatin receptor 2A. FGFR1/FN1 gene fusions can be found in as many as half of cases [50]. Uncertainty remains whether tumors lacking classical PMT features should be included in the same category of neoplasm. Malignant PMTs are rare and most often develop in lesions that have recurred locally and resemble an undifferentiated spindle cell sarcoma (malignant fibrous histiocytoma or fibrosarcoma-like).

## 6. Medical Treatment

### 6.1. Conventional Treatment

Until the tumor is found and resected, medical therapy consists of oral phosphate supplements and calcitriol to aid mineralization in the bone and alleviate the patient’s symptoms. The goal should be to increase phosphate level to >2–2.5 mg/dL, to heal osteomalacia and restore phosphate in the body, especially intracellularly. Both drugs are administered in multiple daily doses, i.e., phosphate at 1–2 g/day and calcitriol at 1–3 mcg/day. Because of their mechanism and due to the underlying disease, patients may develop secondary or tertiary hyperparathyroidism in the long term [51]. This is presumably due to reduced calcitriol levels, or because of the mechanism by which phosphate supplements intermittently reduce ionized calcium. When oral phosphate is administered, it lowers ionized calcium, which stimulates the parathyroid cells to gradually proliferate and eventually become autonomous. This justifies treatment with both calcitriol and phosphate supplements, which should be carefully balanced.

### 6.2. Novel and Potential Treatments for Tumor-Induced Osteomalacia

Burosumab is a human monoclonal antibody against FGF-23, thus counteracting FGF-23 action. Burosumab was approved on 18 June 2020 by the FDA for the treatment of TIO following two clinical studies [52,53]. Burosumab normalizes phosphate levels if the tumor is not resectable or unlocalized, or when it recurs after surgery. During treatment with burosumab, FGF-23 is not overall reduced, but its action on its receptor FGFR1 is prevented by binding to the antibody. This enables the kidneys to restart phosphate reabsorption and reduce the systemic skeletal burden of PMT, after normalizing serum phosphate levels. Briefly, burosumab improves osteomalacia, fracture healing and quality of life in patients with TIO. In the pivotal trial, the drug was able to restore and maintain serum phosphate levels for 144 weeks, and fracture healing improved by 33% over the same period [52,53]. Also, bone pain decreased, and functional mobility improved. Although promising in TIO, burosumab does not halt tumor progression. Of note, measuring FGF-23 levels in patients on burosumab adds no information, as current available kits for measuring FGF-23 cannot yet distinguish between burosumab-bound FGF-23 and free FGF-23 [54].

Infigratinib, an FGF (Fibroblast Growth Factor) receptor 1–3 inhibitor, was used in the trial NCT03510455 for non-resectable or non-localized PMTs. However, due to ocular adverse events, it was immediately abandoned.

Trametinib, a MEK1/2 inhibitor, is a potential agent for treating TIO, although it has not yet been tested on PMTs. The drug was given to a child with NRAS-related cutaneous skeletal hypophosphatemia syndrome and chylothorax. This child had severe hypophosphatemia unresponsive to oral supplementation. Trametinib was able to correct phosphate levels. Regression of recurrent chylothorax and mitigation of skin manifestations were also obtained. This treatment also resulted in an improvement of bone mineral density. Minor adverse events included a transient increase in muscular creatine phosphokinase and serum potassium levels [55].

Other treatments are currently being studied in silico to detect potential molecular targets which halt ligand–receptor interactions, for example, involving α-Klotho, a co-receptor of FGF-23 [42]. For example, ZINC12409120 was shown via in silico docking to block the interaction between Klotho and FGF-23. This compound could be particularly helpful in treating PMTs where no genetic rearrangement of FN1-FGFR1 and/or FN1-FGF1 is identified, but which express elevated levels of Klotho [56].

## 7. Conclusions

PMTs are rare neoplasms which can present with phosphate metabolism alterations or not. These tumors are often difficult to find radiologically and surgery represents a therapeutic choice with curative intent. A cure is less likely when the neoplasm infiltrates the surrounding tissues. In this circumstance, multiple surgeries, the maintenance of medical treatments or both are needed in the long term. A recently developed medication, burosumab, might be considered to improve patients’ quality of life and to heal the underlying osteomalacia in patients with unresectable or recurrent FGF-23-producing PMTs.

## Figures and Tables

**Table 1:** Biochemistry in phosphaturic mesenchymal tumors with concomitant TIO.

Laboratory Parameter	Expected Alteration
Serum phosphate	Decreased
Urinary phosphate	Inappropriately normal or increased in relation to low serum phosphate
Serum calcium	In the lower half on the normal range or slightly decreased
Serum alkaline phosphatase (ALP)	Increased
FGF-23 (intact)	Increased or inappropriately normal
FGF-23 (C-terminal)	Increased
1,25(OH)_2_ vitamin D	Decreased or inappropriately normal in relation to low serum phosphate
25(OH) vitamin D	Variable. It depends on dietary intake and sun exposure
TmP/GFR ^a^	Decreased
FEP	Increased

^a^ TmP/GFR should only be calculated when hypophosphatemia is confirmed on two occasions. Abbreviations: FEP, fractional excretion of phosphate (see text for its calculation).

## Data Availability

Not applicable.
